# Mental Health Service Utilization among Students and Staff in 18 Months Following Dawson College Shooting

**DOI:** 10.3934/publichealth.2014.2.84

**Published:** 2014-04-29

**Authors:** Paule Miquelon, Alain Lesage, Richard Boyer, Stéphane Guay, Pierre Bleau, Monique Séguin

**Affiliations:** 1 Department of Psychology, Université du Québec à Trois-Rivières, 3351, boul. des Forges, C.P. 500, Trois-Rivières, QC G9A 5H7; 2 Institut universitaire en santé mentale de Montréal, 7401, rue Hochelaga, Montréal, QC H1N 3M5; 3 Department of Psychiatry, Université de Montréal, C.P. 6128, succursale Centre-ville, Montréal, QC H3T 1J4; 4 Trauma Studies Centre, Institut universitaire en santé mentale de Montréal, 7401, rue Hochelaga, Montréal, QC H1N 3M5; 5 Department of Psychiatry, Royal Victoria Hospital, McGill University Health Centre, 687 Pine Avenue West, Montreal, QC H3A 1A1; 6 Department of Psychology, Université du Québec en Outaouais, Case postale 1250, succursale Hull, Gatineau, QC J8X 3X7

**Keywords:** school shootings, service utilization, health services, mental disorders, internet use

## Abstract

**Objectives:**

The aim of this study was to investigate service utilization by students and staff in the 18 months following the September 13, 2006, shooting at Dawson College, Montreal, as well as the determinants of this utilization within the context of Canada's publicly managed healthcare system.

**Methods:**

A sample of 948 from among the college's 10,091 students and staff agreed to complete an adapted computer or web-based standardized questionnaire drawn from the Statistics Canada 2002 Canadian Community Health Survey cycle 1.2 on mental health and well-being.

**Results:**

In the 18 months following the shooting, there was a greater incidence and prevalence not only of PTSD, but also of other anxiety disorders, depression, and substance abuse. Staff and students were as likely to consult a health professional when presenting a mental or substance use disorder, with females more likely to do so than males. Results also indicated that there was relatively high internet use for mental health reasons by students and staff (14% overall).

**Conclusions:**

Following a major crisis event causing potential mass trauma, even in a society characterized by easy access to public, school and health services and when the population involved is generally well educated, the acceptability of consulting health professionals for mental health or substance use problems represents a barrier. However, safe internet access is one way male and female students and staff can access information and support and it may be useful to further exploit the possibilities afforded by web-based interviews in anonymous environments.

## 1. Introduction

School shootings have been on the rise in the Americas over the past two decades. These events tend to leave a deep mark on the public imagination. Columbine and Virginia Tech are names associated today with public disasters on a par with natural catastrophes. In the province of Quebec (Canada), the Montreal massacre at the École Polytechnique in 1989 where 11 women were killed by a lone suicide gunman has left as vivid a memory as the Ice Storm of 1998 that left thousands of people blacked out for weeks. It is recognized that the psychological impact of public disasters affecting large populations is much heavier than the physical effects they might have. This is why all current disaster plans comprise psychosocial measures and interventions [1]. The literature examining the aftermath of natural disasters such as hurricanes and earthquakes indicates that in publicly managed systems of care the ensuing surge in requests for psychological services is met by regular resources [2] and that; consequently, the severely mentally ill do not fall by the wayside [3].

There is very little literature available to provide specific guidance in designing disaster plans in response to school shootings. To our knowledge, very little research has investigated the Montreal massacre [4]. This is somehow surprising given that the event took place in a large research-oriented university, although the fact that the event was highly traumatic for the entire Quebec university community might explain this. Similarly, no systematic research of the Columbine shooting was undertaken and reported in the scientific literature. However, a number of articles have been published in the scientific literature concerning the Virginia Tech shooting, where staff and students were more forthcoming. First, *Disaster Medicine and Public Health Preparedness* put out a special issue in short order containing a series of experience-based summaries along with recommendations, including on how to improve national disasterplans [5],[6]. However, the focus was on the response to medical needs, with little or no consideration given to the response to mental or behavioral health needs [7],[8]. More recently, articles have been published concerning the reaction of subgroups immediately after exposure to the event [9] and one year later at Virginia Tech. These articles report severe behavioral reactions, including post-traumatic disorders, depression and service needs that persist well past the first few weeks [10],[11].

The opportunity to document the psychological impact of and behavioral reactions to the September 13, 2006, shooting at Dawson College, Montreal, and the ensuing utilization of services arose one year after the event. At that moment, Quebec Government commissioned a study [12]–[15] at the request of Dawson College and the McGill University Health Centre, which provided the first medical disaster response and played the leading role over the next two years delivering psychological and/or behavioral interventions. Recommendations and a proposed multimodal intervention plan have already been published [16]. This article, instead, will report in greater detail on service utilization by students and staff in the 18 months following the shooting and the determinants of this utilization within the context of Canada's publicly managed healthcare system.

In Canada and Quebec, all medical healthcare, including hospital care and mental health care, is covered under a universally accessible, publicly managed system of care. In Quebec, the system covers medication as well. Psychotherapy, however, is limited to the severely mentally ill and otherwise difficult to access in the public system. Psychologists in private practice offer psychotherapy paid for by private insurance or out of pocket. In 2002, the Canadian Community Health Survey cycle 1.2 (CCHS 1.2) on mental health and well-being [17] showed that general practitioners were the health professionals most often consulted for reasons of mental health. In Quebec, psychologists and psychiatrists came in second and third, respectively. This was not much different from the situation in the United States [17] or in French-speaking European countries, though the utilization of general practitioners was greater in Canada [18]. Still, no more than half of the people with a mental disorder consulted a health professional, though women, middle-aged people and native people were more likely to do so [17]. As it turned out, acceptability proved much more of a barrier to consulting than accessibility did [19], a finding echoed in studies conducted in other industrialized countries [27].

Within this context, the aim of this study was to investigate service utilization by students and staff in the 18 months following the September 13, 2006, shooting at Dawson College, Montreal, as well as the determinants of this utilization within the context of Canada's publicly managed healthcare system. It was hypothesized that, following the Dawson College shooting, we would observe a surge in psychological distress, which would influence the utilization of publicly managed care and community, school and private services for mental health reasons. A retrospective design was used to verify these assumptions.

## 2. Materials and Method

### 2.1. Population and sampling

The population studied was composed of all the students (n = 8,779) and employees (n = 1,312) of Dawson College on September 13, 2006. The research plan specified a simple random sample of 2,000 respondents which proved to be impossible to achieve. In the end, a total of 948 respondents filled in the survey, 854 students and 94 employees. As this was a convenience sample not entirely representative of the study population, the respondents were weighted to reflect the Dawson College population in terms of the ratio of males to females (sex) and of students to employees (status). Considering that a simple random sample of 2,000 respondents was initially contacted, the response rate could be estimated to 47%. Since the response rate found in research on population's health conducted by national institute like Statistic Canada is around 70% (e.g., the response rate for the 2002 Canadian Community Health Survey was 77% whereas the response rate for the 2012 Canadian Community Health Survey was 69%), a higher response rate was expected. This modest response rate was explained by the fact that the data gathering was conducted at the end of the semester and that many did not wish to revisit the tragic event more than 18 months later.

### 2.2. Procedures

At the beginning of April 2008, a letter was sent by mail presenting the study's goals and indicating that a member of the research team would phone to solicit their participation and to answer any questions. After this initial contact, participants received a complete description of the study and were asked to sign a written or web-based inform consent. They were then invited, until end of June 2008, to complete a computer-administered questionnaire either in a room set up specifically for the purpose on campus (28%) or online from home on a secure website. The project was approved by the research ethics committees of the McGill University Health Centre, Dawson College and the Centre de recherche Fernand-Seguin (now Research Centre of the Institut universitaire en santé mentale de Montréal).

### 2.3. Measuring instruments

The data were collected via an instrument developed specifically for the purposes of our study. It was constructed from several validated questionnaires. Only those components of the instrument for which results are presented here are described below. The collection of data on psychological signs and symptoms made it possible to determine whether such signs and symptoms preceded or followed exposure to the potentially traumatic event. The researchers therefore obtained estimates of the prevalence (active cases in a given population) and incidence (new cases in that population) of the various disorders studied following the event.

#### 2.3.1. Composite International Diagnostic Interview (CIDI)

The prevalence of mental disorders before and after September 13, 2006, and the incidence of these disorders after the shooting were estimated with the help of a computerized bilingual version of the Composite International Diagnostic Interview (CIDI) for the Diagnostic and Statistical Manual of Mental Disorders IV (DSM-IV). The CIDI version used for this investigation (CIDI/Dawson) was constructed from the one used by Statistics Canada in the 2002 CCHS 1.2 on mental health and well-being and the CCHS Canadian Forces Supplement [20]. The CIDI/Dawson version covered major depressive episodes, the main anxiety disorders (post-traumatic stress disorder, social phobia, agoraphobia with or without panic disorder), and the disorders associated with substance, alcohol and drug abuse and dependence.

#### 2.3.2. Mental Health Services

Use of health services for mental health reasons in the year before and after the shooting was estimated with the Mental Health Services and Psychotropic Medication components of the CCHS 1.2 questionnaire (20). The components covered utilization, sites, costs and satisfaction regarding services put in place specifically after the shooting. The results of the 2002 CCHS 1.2 for Quebec regarding services were published by the Institut de la statistique du Québec [21],[22]. They were used as a reference index for the Dawson study results.

## 3. Results

### 3.1. Sociodemographic and sociomedical characteristics

Table 1 presents the distribution of respondents by main sociodemographic and sociomedical characteristics. The vast majority of respondents were students (90%), while employees made up close to 10%. This distribution was consistent with that of the Dawson College studied population, which was composed of 8779 students (about 87% of the studied population) and 1312 employees (about 13% of the studied population). As expected for the student subgroup, close to 45% of the respondents were 19 years old or under and just over half (52%) were 20 to 34 years of age at time of shooting. The mean age of the students was 21.3 years (±5.07). Almost 90% of the employees were 35 years old or over and their mean age was 50.5 years (±12.36). The age difference between students and employees was significant (t = -22.81; *p* < 0.0001). This distribution was also consistent with that of the Dawson College studied population since there was 43% of men and 57% of women among students and 46% of men and 54% of women among employees. Moreover, the mean age of students from Dawson College was 21 years while it was 50 years for employees.

**Table 1.** Sociodemographic and sociomedical characteristics of sample drawn from population of students and employees of Dawson College as at shooting on September 13, 2006 (data weighted by sex and age).

Characteristics	n	%
**Status at Dawson College**		
Students	854	90.1
Employees	94	9.9
**Sex - students & employees**		
Male	407	42.9
Female	542	57.1
**Sex - students**		
**Male**	364	42.6
**Female**	490	57.4
**Sex - employees**		
**Male**	43	45.5
**Female**	51	54.5
**Age groups – students (years)**		
≤19	380	44.5
20–34	444	52.0
≥35	30	3.5
**Age groups – employees (years)**		
≤19	3	3.6
20–34	6	6.7
≥35	84	89.6
**Marital status - students & employees**		
Single	772	84.4
Married	94	10.4
Common-law	32	3.5
Divorced	17	1.9
*Missing data*	(34)	
**On site at time of shooting – students and employees**		
Present	727	78.8
Absent	196	21.2
*Missing data*	(26)	
**Current Mental health perception - students & employees**		
Excellent	184	19.7
Very good	340	36.4
Good	256	27.4
Fair	120	12.8
Poor	35	3.7
*Missing data*	(13)

Regarding current self-perceived mental health, a majority of respondents (56%) reported it to be excellent or very good, while 17% estimated it was barely average or poor. Males and employees evaluated their mental health slightly more positively (χ^2^ = 18.6, *p* < 0.001 and χ^2^ = 20.5, *p* < 0.001, respectively). Interestingly, a lower proportion of the Dawson College population reported excellent or very good self-perceived mental health compared with the general young adult population of Quebec (74%) in the 2002 CCHS 1.2.

### 3.2. Incidence and prevalence of mental disorders

Table 2 presents the incidence of the various mental disorders selected for our investigation. These were new cases observed in the sample after the shooting on September 13, 2006, and did not include cases that appeared before that date. The prevalence of the disorders in the 18 months after the shooting is also reported. No data were available on the incidence of these disorders in the Quebec general population, but the CCHS 1.2 (23) reported past-year prevalence against which the 18-month Dawson data could be compared. When 18-month post-event prevalence of all disorders was considered, it proved 2 to 3 times as great as for the general population for all disorders related to anxiety, depression and substance abuse. Almost one half of this surge in disorders was due to incident cases that occurred for all types of disorders, in particular depression and substance abuse.

**Table 2.** Prevalence and incidence of mental disorders in 18 months following Dawson College shooting measured with adapted Composite International Interview Schedule *(data weighted by sex and age)*.

Characteristics	%		CI95%
**Major depression episode**			
Prevalence	12.1		10.0–14.2
Incidence	5.0*		3.5–6.5
**Alcohol dependency**			
Prevalence	8.7		6.8–10.6
Incidence	4.7*		3.3–6.1
**Social phobia**			
Prevalence	9.6		7.6–11.6
Incidence	3.4*		2.1–4.7
**Posttraumatic stress disorder**			
Prevalence	3.4*		2.2–4.6
Incidence	1.8**		0.9–2.7
**Illicit Drug dependency**			
Prevalence	2.6*		1.6–3.6
Incidence	0.9**		0.3–1.5
**Agoraphobia**			
Prevalence	3.0*		1.9–4.1
Incidence	0.4**		0.0–0.8
**Panic disorder**			
Prevalence	1.9*		1.0–2.8
Incidence	0.3**		0.0–0.7
**Agoraphobia without panic disorder**			
Prevalence	2.1*		1.2–3.0
Incidence	0.1**		0.0–0.3
**Presence of at least one mental disorder**			
Prevalence	30.9		27.6–34.1
Incidence	18.1		15.1–21.1

* Coefficient of variation between15% and 25%; interpret with caution; ** Coefficient of variation greater than 25%; estimate given only as an indication.

### 3.3 Service utilization

Table 3 presents the prevalence of resource utilization in the Dawson College population after the shooting. Services were divided into three general sectors or categories, namely: volunteer (i.e., religious or spiritual counsellor, internet, crisis hotline, and help group), professionals (i.e., psychiatrist, general practitioner, psychologist, substance abuse counselor and other professional or responder) and hospital (i.e., hospitalization and emergency room). A number of differences emerged relative to utilization in the general populations of Quebec and Canada in 2002. Prevalence in our study population was twice as high for psychiatrists, 50% greater for psychologists, and immensely higher regarding internet use, where the numbers were 14% vs. 0.5%. Because of this internet utilization, prevalence in the volunteer sector and overall utilization categories was five and two times as great as in the general population, respectively, whereas the professionals and hospital sectors fell within the 95% confidence intervals of both our study and the CCHS 1.2.

**Table 3.** Prevalence of use of various behavioral health services in Dawson College population in 18 months following shooting September 13, 2006 *(data weighted by sex and age).*

Resource used	Prevalence	
	%	CI95%
Psychiatrist	5.8	4.2–7.4
General practitioner	6.6	4.9–8.3
Psychologist	6.6	4.9–8.3
Substance abuse counselor	0.4**	0.0–0.8
Other professional or responder	1.3**	0.5–2.1
***At least one of the above professionals***	**13.0**	**0.7–15.3**
Hospitalization	0.5**	0.0–0.0
Emergency Room	0.3**	0.0–0.7
***At least one of the above professionals or hospital services***	**13.2**	**10.9–15.5**
Religious or spiritual counsellor	1.0**	0.3–1.7
Internet	14.1	1.7–16.5
Crisis hotline	0.9**	0.3–1.5
Help group	1.0**	0.3–1.7
***At least one of these volunteer sector resources***	**I5.7**	**13.2–18.2**
***At least one of the 11 resources above***	**23.0**	**20.1–25.9**

* Coefficient of variation between15% and 25%; interpret with caution; ** Coefficient of variation greater than 25%; estimate given only as an indication.

To evaluate the determinants of post-event service utilization according to Andersen's classic model (see also 17 for the CCHS 1.2 analysis of determinants), we used multivariate analyses in order to take into consideration simultaneously all of the participant characteristics selected for our study. Table 4 presents the significant multivariate associations between prevalence of service utilization after the shooting and, respectively, sociodemographic indicators (e.g., sex and age) and prevalence of the various mental disorders selected for investigation (e.g., prevalence of major depressive episode in 18 months after shooting)

An analysis was run on three models of services: 1) all services (general practitioners, mental health specialists, other professionals, hospital sector and volunteer services); 2) all professionals; and 3) internet-based services. Only major depression and social phobia proved determinants in the three models. Status at Dawson College (student or employee) and panic disorder showed no influence once other variables were considered. Both sex and agoraphobia influenced help seeking in models 1 and 2 (all services and professionals only). Age influenced only internet service utilization. Alcohol dependence had an effect only on seeking help from professional providers. Drug dependence and PTSD had no effect in this model but did in models 1 (all services) and 3 (internet services).

Moreover, an additional multivariate analysis considering incidence rather than prevalence of the various mental disorders selected for this investigation showed a similar pattern of results. More specifically, major depression proved a determinant in the three models, sex influenced help seeking in models 1 and 2 (all services and professionals only), and social phobia influenced help seeking in models 1 and 3 (all services and internet use). Drug dependence influenced only internet service utilization. However, age, status at Dawson College, incidence of alcohol dependence, PTSD, agoraphobia, and panic disorder did not prove determinants in any of the three models.

Finally, the main reason for not consulting given by people who had at least one mental health problem post event was that they preferred to “figure things out on their own”. In this regard, the respondents in our study did not differ from those in the 2002 CCHS 1.2 (Quebec), where the majority of people with a mental disorder did not consult. The main reason for not using services, then, had to do more with acceptability than accessibility [21].

## 4. Discussion

Our results corroborate the known determinants of service utilization, namely, presence of mental disorder, substance dependence, and sex. However, in this generally well educated population, students and employees did not show a differential tendency to consult when their other characteristics were controlled. Moreover, all disorders–and not only PTSD–led respondents to seek help. Interestingly, in the case of internet services, which stood out for the magnitude of the increase following the shooting, sex proved neither a predictor nor a determinant of use. Moreover, though students were more likely than older employees to use the internet, the latter still made ample use of it. This suggests that this means of communication can help overcome the reluctance in males to seek help when experiencing mental health or substance use problems.

**Table 4.** Multivariate associations across three models of service utilization after September 13, 2006, and prevalence of various mental disorders and sociodemographic indicators, along with interpretation of odds ratio *(data weighted by sex and age).*

Characteristic	**Overall service utilizationin 18 months post shooting** Participants used at least one of these resources: psychiatrist, general practitioner, psychologist, substance abuse counselor, religious or spiritualcounselor, other professional or responder, hospitalization, Emergency room, internet, crisis hotline, help group		**Professional resources usedin 18 months post shooting** Participants consulted at least one of these five professionals: psychiatrist, general practitioner, psychologist, substance abuse counselor, other professional		**Internet utilizationin 18 months post shooting** Participants used internet		
	Adjustedodds ratio	95% CI	Adjustedodds ratio	95% CI	Adjustedodds ratio		95% CI
**Sex**	1.9	1.2–2.9 (more women used these services)	3.0	1.6–5.6	NS		NS
**Female**							
Male*							
**Age (years)**	NS	NS	NS	NS	0.2 (younger people used Internet more)		0.2–0.8
≤ 19*							
20–34							
≥ 35							
**Status at Dawson**	NS	NS	NS	NS	NS		NS
Student*							
Employee							
**Prevalence of major depressive episode 18 months post shooting**	7.2	3.9–13.2 (if depression present, more likely to use services overall)	2.9	1.5–5.9	7.0		3.7–13.4
No*							
Yes							
**Prevalence of alcohol dependence 18 months post shooting**	NS	NS	2.9	1.4–5.9 (if alcohol dependent, more likely to use professional services)	NS		NS
No*							
Yes							
**Prevalence of social phobia 18 months post shooting**	2.5	1.3–4.7 (if social phobia present, more likely to use services)	2.9	1.4–5.8	2.7		1.4–5.2
No*							
Yes							
**Prevalence of post-traumatic stress disorder 18 months post shooting**	6.2	1.5–26.3 (if PTSD present, more likely to use services)	NS	NS	6.2		1.7–22.1
No*							
Yes							
Prevalence of drug dependence 18 months post shooting	7.9	2.1–29.6 (if drug dependence present, more likely to use services)	NS	NS	7.0		1.9–26.5
No*							
Yes							
**Prevalence of agoraphobia 18 months post shooting**	7.4	1.7–31.7 (if agoraphobia present, more likely to use services)	4.4	1.2–16.5	NS		NS
No*							
Yes							
**Prevalence of panic disorder 18 months post shooting**	NS	NS	NS	NS	NS	NS	
No*							
Yes							

Reference category. Note. For internet use, the adjusted odds ratio of 0.2 indicates that the utilization level of the age group ≥ 35 years was only 20% of the level of the age group ≤ 19 years.

The traumatic stress responses following the Dawson College shooting included not only PTSD, as expected, but also a variety of anxiety disorders, major depression and alcohol abuse. Over two-thirds of those affected had experienced these common mental disorders in the past, which suggests that those with a pre-existing vulnerability are less able to cope [24]. As hypothesized and reported in Table 3, services were used for mental health reasons, especially specialized services (psychiatrists and psychologists), on a par with general practitioners who were, as is usually the case in Canada and Quebec's publicly managed healthcare system, the service providers most often consulted for this purpose. However, only 13% of the sample saw at least one of these professionals, which does not differ much from the 10% registered for the general population in 2002. This is reminiscent of what has emerged from recent mental health community surveys to the effect that the majority of people with common mental disorders do not seek care and that acceptability more often than accessibility is given as the reason for this [17],[18],[25]. Nevertheless, we consider that this result principally indicates or outlined a need to educate university staff and students on when to seek care (e.g., that figuring it out alone is not always the solution), rather than a judgment on seeking care.

The considerable internet use for mental health reasons by students and staff (14% overall) was surprising. This was the driver that doubled overall service utilization compared with the results of the CCHS 1.2. Unfortunately, we did not investigate the specific nature of this utilization and cannot ascertain from our questionnaires whether platforms like Facebook were used to create chat rooms. However, such chat rooms were reported in focus groups we conducted, with participants confirming they were all spontaneous and with no official employee, student or college input or obstruction [15].

As highlighted by Séguin and colleagues [16], the fact that students and employees with psychological distress used the internet as a resource to gain information on mental health issues and services suggests that the majority of these individuals might have been willing to consult had there been a link on the websites visited leading to other sites offering mental health services. Therefore, the accessibility and availability of social network platforms should be key elements to consider in designing a multimodal intervention protocol for responding to crisis situations after the fact. Clearly, the issue warrants further research in the interest of the general population.

The biggest limitation of the study has to do with the representativeness of our volunteer convenience sample of 948 respondents or about 10% of the Dawson College population of students and staff. Arguably, the people who participated in the study might have been those more deeply affected by the event. However, we evidenced in our sample a reluctance to engage in treatment or in help seeking comparable to that observed in recent population surveys like the CCHS 1.2 on mental health. In addition, our sample does have the merit of presenting a staff-student and male-female split comparable to that in the Dawson College population. Another important limitation of this research regards the reliability of the estimates, which can have implications on the findings. Thus, similar future studies, that either use a different design or recruit a larger population, are needed.

College directors indicated that, at 18 months post event, the community was well into a closure and recovery phase. This suggests that conducting the study earlier would have afforded a better window of opportunity for participation. Unfortunately, the lag was due to funding delays, time required to develop the web-based questionnaire, and the ethics board authorization process. On the upside, the systematic retrospective assessment of disorders and service utilization over a longer period gives indications on long-term effects. It also provides an insight on the persons most affected by the event, who turned out to be those with a greater vulnerability, prior disorders and prior experience with services. However, further analyses demonstrating that those who responded to the survey had more severe mental health outcomes than those who did not would be necessary to support this assumption.

Finally, another potential limitation of this study is that results on mental health and service utilization are compared to findings of the 2002 CCHS 1.2 for Quebec. However, at the time the article was revised, the 2012 CCHS data regarding mental and service use were not published yet for the province of Quebec. Therefore, it was difficult to use more recent findings, obtained at the provincial level, as a reference index for the Dawson study results.

## 5. Conclusions

The results from our study point to unmet needs for mental health care on a par with what is observed in the Canadian and Quebec general population. The disorders requiring treatment were common mental disorders like depressive and anxiety disorders and substance abuse. As is the case in the general population, treatment acceptability was a major issue. Where public health in Quebec is concerned, two recommendations are put forth, namely, 1) that efforts be made to promote mental health literacy in schools and the media and 2) that workplaces, including schools, adopt healthy work environment standards, including detection of and response to distress, common mental disorders and substance abuse, and the creation of an environment more receptive to mental health prevention, treatment and rehabilitation [25], [26].
